# Editorial: Water and carbon dynamics, ecosystem stability of forest and grassland in response to climate change

**DOI:** 10.3389/fpls.2024.1306381

**Published:** 2024-04-03

**Authors:** Hui Huang, Yu Zhou, Xiaoming Kang, Xianjin Zhu, Xiaojuan Tong

**Affiliations:** ^1^ Key Laboratory of Tree Breeding and Cultivation of National Forestry and Grassland Administration, Research Institute of Forestry, Chinese Academy of Forestry, Beijing, China; ^2^ Collaborative Innovation Center for Sustainable Forestry in Southern China, Nanjing Forestry University, Nanjing, China; ^3^ Henan Xiaolangdi Forest Ecosystem National Observation and Research Station, Jiyuan, China; ^4^ Academic Affairs Office, Jining Polytechnic, Jining, China; ^5^ Institute of Wetland Research, Chinese Academy of Forestry, Beijing, China; ^6^ College of Agronomy, Shenyang Agricultural University, Shenyang, China; ^7^ School of Ecology and Nature Conversation, Beijing Forestry University, Beijing, China

**Keywords:** water and carbon cycling, climate change, ecosystem fluxes, ecosystem stability, forest and grassland ecosystem

## Introduction

Climate change, characterized by long-term trends in temperature and rainfall, has emerged as a prominent concern in recent years (Seddon et al., 2016), exerting substantial impacts on the global carbon, water, and energy cycles of forest and grassland ecosystems. Additionally, the increasing frequency of extreme weather events can have devastating consequences for various terrestrial ecosystems (IPCC, 2023).

To further investigate the impact of climate change on forest and grassland ecosystems and to support China’s efforts to reach its peak carbon dioxide emissions and carbon neutrality goals, this Research Topic was proposed. This Research Topic comprises 23 original research articles and 1 opinion article, presenting recent advancements in the following areas: (1) carbon, water, and energy cycling of forest and grassland ecosystems in response to climate change, and (2) the response and adaptation of vegetation characteristics and ecosystem stability to climate change.

## Carbon, water, and energy cycling in forest and grassland ecosystems

Carbon storage in terrestrial ecosystems plays a crucial role in mitigating climate change and achieving the goal of “carbon neutrality” by 2060. Chen et al. quantified the dynamics of soil organic carbon density (SOCD) from 1982 to 2020 and identified the dominant drivers using random forest regression. The authors identified temperature, vegetation greenness, elevation, and wind speed as the primary drivers of changes in SOCD. Grassland degradation may contribute to the loss of soil organic carbon and have a negative impact on the climate. This emphasizes the importance of prioritizing carbon management. Based on inventories conducted in the Taihang Mountains Priority Reserve, Lu et al. found that structural diversity enhanced carbon storage in forests, while species diversity promoted carbon storage in shrublands. In addition, increasing elevation strengthened the relationship between structural diversity and carbon in forests, but weakened the relationship between species diversity and carbon in shrublands. All of these differences were attributed to niche and architectural complementarity, in addition to life strategies that could guide differentiated management of forests and shrublands. Liu et al. synthesized field measurements of ecosystem carbon use efficiency (CUEe) and demonstrated significant differences in CUEe among different ecologically vulnerable areas. This research is valuable for understanding the impact of climate change on vegetation.

Evapotranspiration (ET) is a crucial factor in regulating global water and energy cycles. Based on the annual evapotranspiration (AET) values of forests and grasslands in China measured using eddy covariance, Fan et al. analyzed the differences in AET values and spatial variations between forests and grasslands. They found that forests had significantly higher AET values than grasslands. However, there was no significant difference in AET values between forests and grasslands after controlling for mean annual precipitation (MAP)-related factors. The spatial variation of AET in forests and grasslands is influenced by multiple factors, with MAP playing a dominant role. Furthermore, AET exhibited similar responses to MAP in different ecosystem types, resulting in comparable AET values under similar climatic conditions.

## Vegetation response and adaptation to climate change

The Qinghai-Tibetan Plateau (QTP) is the highest and largest plateau in the world, characterized by diverse topographic conditions and climatic types. It experiences relatively little disturbance from human activities, making it an important area of research for studying vegetation response and adaptation to climate change. Using the MaxEnt model, Hu et al. simulated the potential impacts of climate change on the richness and distribution of endangered orchid species in the 2050s and 2070s. They found that the potential distribution area of orchid species would increase, while the richness of orchid species would generally decrease with increasing elevation in the future. Additionally, the selection of protected areas in the southeastern QTP should be a key consideration for future conservation plans. These results help us to predict the potential impacts of climate change on species richness and distribution, which are crucial for the conservation of endangered species. He et al. measured leaf chlorophyll (Chl) levels of grassland species at various sites in the QTP and identified a significant spatial pattern in leaf Chl. They observed that leaf Chl decreased with latitude but increased with longitude, under the combined effect of climate (photosynthetically active radiation (PAR), humidity index), soil nutrients, and plant functional group. Moreover, plant evolution has played a dominant role in shaping the variation in leaf Chl levels. It was observed that leaf Chl levels decrease non-linearly with plant evolutionary divergence time, which corresponds well with the non-linearly increasing trend in PAR or the decreasing trend in temperature during the geological timescale uplift of the Tibetan Plateau. This finding has the potential to prompt a reevaluation of the photosynthetic capacity of plants and the carbon cycle from an evolutionary perspective.

In recent years, there has been an increase in the frequency and duration of droughts. Also, the impact of these drought events on carbon fluxes has received considerable attention, especially in the mid-latitude semi-arid region. Qian et al. reported findings that the regulation of soil respiration by drought depends on seasonal timing and communities in a semi-arid grassland. The prolonged drought had more pronounced effects on soil respiration and heterotrophic respiration than the initial drought. Under drought conditions, soil water content indirectly regulates soil respiration through the microbial biomass content and gross primary production. More attention should be paid to droughts with different seasonal timing and the consequent changes in plant structure when predicting carbon dynamics under climate change. Jia et al. investigated the growth resilience indices and intrinsic water use efficiency (iWUE) of *Q. variabilis* and *R. pseudoacacia* across three crown classes using dendrochronology and carbon isotopes. Growth resilience indices, iWUE, and the current canopy health score, which serves as a proxy for vulnerability to canopy dieback, were analyzed to assess the relationships between these indicators and drought-induced mortality. The study provides helpful information on species selection and management measures for plantations in lithoid mountain areas with an increasing risk of drought. Based on eddy covariance fluxes from different ecosystems, Liu et al. found that soil factors have a greater impact on carbon fluxes in the drylands than climate and vegetation factors. It is necessary to fully consider the disparate effects and cascading relationships of these three factors on fluxes.

Moreover, frequent temperature extremes are common in the mid- and high-latitudes of the Northern Hemisphere, which is also an important area of research for climate change. Yan et al. demonstrated that temperature is the primary factor influencing seasonal fluctuations in net ecosystem carbon exchange on a daily scale, based on 11 years of eddy covariance observations. They also found that extreme temperature events would lead to a significant reduction in carbon uptake in the boreal forest ecosystem. These findings provide valuable data for assessing carbon budgets in the context of climate change.

## Perspective

This Research Topic presents the latest research on how climate change and extreme weather events affect the mass and energy cycles in forest and grassland ecosystems and the adaptation of plants. It highlights the importance of ecosystem management and provides insights into evolutionary processes, species diversity, plant functional groups, and the impact of drought at different times of the year. The Research Topic also encompasses the interconnected relationships among these factors. However, the coverage of this research in terms of time and space is still limited. More research is still needed to gain a deeper understanding of how ecosystems interact with and respond to climate change.

## Author contributions

HH: Writing – original draft, Writing – review & editing. YZ: Writing – original draft, Writing – review & editing. XK: Writing – review & editing. XZ: Writing – original draft. XT: Writing – original draft.
